# Experimental study on the repair of peripheral nerve injuries *via* simultaneously coapting the proximal and distal ends of peripheral nerves to the side of nearby intact nerves

**DOI:** 10.3389/fneur.2023.1088983

**Published:** 2023-04-06

**Authors:** Dongdong Li, Qi Yang, Xin Liu, Jing Jia, Guangbo Liu, Kewen Bai, Shicheng Jia, Jun Peng, Fei Yu

**Affiliations:** ^1^Department of Orthopedics, Strategic Support Force Medical Center, Beijing, China; ^2^Department of Ultrasonography, Peking University Shenzhen Hospital, Shenzhen, China; ^3^Department of Sports Medicine, Peking University Shenzhen Hospital, Shenzhen, China; ^4^Department of Bone and Joint Surgery, Peking University Shenzhen Hospital, Shenzhen, China; ^5^National and Local Joint Engineering Research Center of Orthopaedic Biomaterials, Peking University Shenzhen Hospital, Shenzhen, China

**Keywords:** peripheral nerve defect, end-to-side anastomosis, functional recovery, effective approach, repair mechanism

## Abstract

**Introduction:**

Peripheral nerve defect is a difficult disease to treat in clinical practice. End-to-side anastomosis is a useful method to treat it. At present, the end-to-side anastomosis method does not involve the proximal nerve, which results in a waste of proximal donor nerves, and even the formation of traumatic neuromas at the proximal end. The patients suffer from traumatic neuralgia and the curative effect is unsatisfactory.

**Methods:**

In this study, an improved end-to-side anastomosis technique was proposed in this study: both the proximal and distal ends of the damaged common peroneal nerve were sutured to an adjacent normal tibial nerve. Moreover, the possible role and mechanism of the proposed technique were explained at the physiological and anatomical levels. In this study, a 10 mm common peroneal nerve defect was made in SD rats, and the rats were randomly divided into three groups. In Group I, the distal end of the common peroneal nerve was attached end-to-side to the fenestrated tibial nerve adventitia, and the proximal end was ligated and fixed in the nearby muscle. In Group II, the tibial nerve adventitia was fenestrated and the epineurial end-to-end anastomosis surgery was performed to suture the proximal and distal ends of the common peroneal nerve. Rats in Group III were taken as control and received sham operation. Twelve weeks after the operation, the recovery of the repaired nerve and distal effector functions were examined by the sciatic functional index, electrophysiology, osmic acid staining, the muscle wet weight ratio, and the muscle fiber cross-sectional area.

**Results:**

It was found that these results in Group II were similar to those in Group III, but better than those in Group I. Through retrograde tracing of neurons and Electrophysiological examination in Group II, the study also found that the proximal common peroneal nerve also could establish a connection with tibialis anterior, even gastrocnemius.

**Discussion:**

Therefore, it is inferred that fostering both the proximal and distal ends of defective peripheral nerves on normal peripheral nerves using the end-to-side anastomosis technique is a more effective approach to repairing injured nerves.

## Introduction

Peripheral nerve injury can cause sensory and motor dysfunction, and delayed treatment will lead to poor prognosis and even lifelong disability (1). According to the severity and nature, peripheral nerve injury can be divided into nerve conduction dysfunction, nerve axon interruption and nerve rupture. After the peripheral nerve ruptures, pathological changes occur in nerve fibers, neuronal bodies and target organs (1–3). At present, common methods for repairing peripheral nerve injuries include neurolysis, nerve suture, nerve transplantation, nerve transfer and nerve implantation. With the development of orthopedics and microsurgery technology, progress has been made toward repairing peripheral nerve injuries (4, 5). However, further improvement of the repair effect is required, especially for large segmental peripheral nerve defects. The interrupted connection between the injured proximal and distal nerve fibers makes the repair more difficult in clinical treatment (6, 7).

One method to repair peripheral nerve injuries is end-to-side anastomosis, which sutures the distal end of the injured nerve to the side wall of the adjacent healthy nerve trunk. In this way, the injured nerve can regenerate and the function of the target organ can restore. Since it was proposed, end-to-side anastomosis has attracted increasing attention (8, 9). However, this repair method has some demerits. The disuse of the injured proximal peripheral nerve gives rise to a waste of donor nerves and proximal traumatic neuromas. The neuromas further result in pain and discomfort, and worsen the repair effect (10). To solve this issue, some scholars have modified the end-to-side anastomosis method, and fostered the injured proximal peripheral nerve on the side wall of an adjacent normal nerve. The updated method was proven effective in repairing injured peripheral nerves (11). Nevertheless, there are few studies on the improved method, so its specific repair effect and possible mechanism require further elaboration.

In view of this, an SD rat model with a large segmental common peroneal nerve defect was established in this paper. Besides, the improved end-to-side anastomosis technique was used to suture both the proximal and distal ends of the damaged common peroneal nerve to an adjacent normal tibial nerve. The functional recovery of the injured common peroneal nerve and its effector 12 week after repairing, and the connection between the repaired nerve and motor neurons in the anterior horn of the spinal cord were examined. The purpose of this study is to preliminarily explain the possible role and mechanism of this method at physiology and anatomy levels.

## Materials and methods

### Animal models

Eight-week-old 150–180 g SD rats were purchased from Beijing Vital River Laboratory Animal Technology Co., Ltd. The operation was carried out following the Guidelines for Ethical Review of Animal Welfare in China (GB/T 35892-2018). All experiments were approved by the Medical Ethics Committee of Peking University People's Hospital (Permit Number: 2020PHC015, 3/1/2019). The rats were anesthetized with sodium pentobarbital intraperitoneally (Merck, Darmstadt, Germany; 30 mg/kg) before random grouping. In Group I (*n* = 9), the distal end of the common peroneal nerve was coapted end-to-side to the fenestrated tibial nerve adventitia, and the proximal end was ligated and fixed in the nearby muscle. In Group II (*n* = 9), the tibial nerve adventitia was fenestrated and epineurial end-to-end anastomosis was performed to anastomose the proximal and distal ends of the common peroneal nerve. Rats in Group III (*n* = 9) were taken as control and underwent sham operation. After awakening, the rats were fed in groups, and food and drinking water were automatically supplied. A 12:12 light/dark natural circle was adopted in the feeding environment.

### Sciatic functional index test

The motor function of rats was measured by the CatWalk XT gait analysis system (Nodus, Netherlands), which can automatically record the motion parameters. The camera was placed at the correct position and corresponding rat parameters were setup. Before recording, the rats were trained to be familiar with the running environment. All rats in different groups were recorded automatically. The calculation formula of sciatic functional index (SFI) is SFI = 109.5 (ETS–NTS)/NTS – 38.3 (EPL–NPL)/NPL + 13.3 (EIT–NIT)/NIT – 8.8. SFI is a reliable method for the assessment of the extent of injury and the degree of recovery of the sciatic nerve of the rat. E: experimental; N: normal; PL: print length from heel to longest toe; TS: total spread, or the transverse distance between the 1st and the 5th toes; IT: intermediate toes, or the transverse distance between the 2nd and 4th toes.

### Neuroelectrophysiological detection

**Figure 1:**
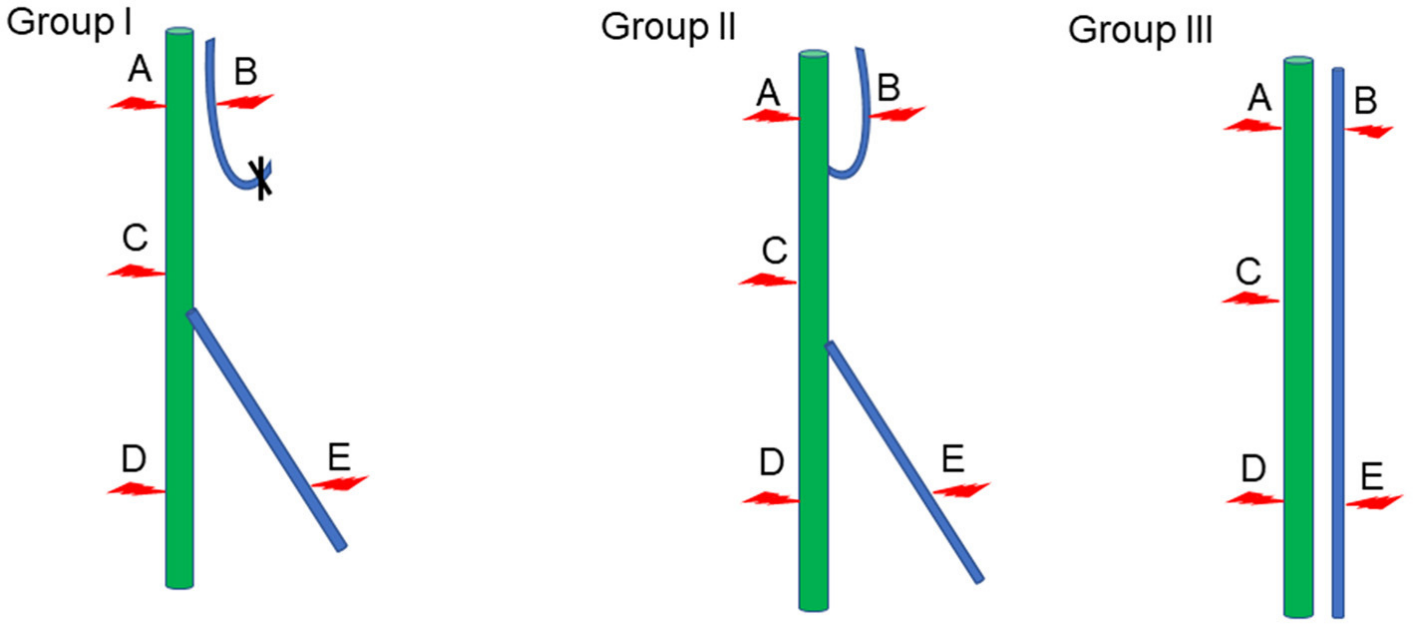
Schematic diagram of electrophysiological detection. Green represents the tibial nerve, blue indicates the common peroneal nerve, and the red arrow denotes the location of the stimulation electrode.

The compound action potential of gastrocnemius and tibialis anterior muscles was detected by the Medlec synergy electrophysiological system (Oxford Instruments Inc., Oxford, UK) in each group. After the rats in each group were anesthetized with sodium pentobarbital, the repaired nerves were exposed. Then the stimulation electrodes were placed at positions A, B, C, D, and E of the nerve (Figure 1), and the electrical signals of gastrocnemius and tibialis anterior muscles were recoded through receiver electrode, separately. In the meantime, the action potentials of gastrocnemius and tibialis anterior muscles recorded at point A were taken as the maximum action potentials of Group I and Group II. The action potential of the gastrocnemius muscle recorded at point A and the action potential of the tibialis anterior muscle recorded at point B were adopted as the maximum action potentials of Group III. Statistical analysis was subsequently made to evaluate the size of the compound muscle action potential (CMAP) of each group. Rectangular pulse (0.1 ms duration, 0.06 mA intensity, 5 Hz frequency) was used to acquire the CMAP.

### Histological observation of the repaired nerve

The tibial nerve and common peroneal nerve 2 mm away from the anastomosed common peroneal nerve were collected. The nerve sample was soaked in 4% paraformaldehyde for 12 h and then dyed with 1% osmic acid for another 12 h. After the sample was dehydrated with 50, 70, 90, and 100% ethanol successively, it was embedded in paraffin, and sliced into a 2 μm-thick piece (Leica RM2135, Wetzlar, Germany). The piece was placed on the glass slide. After sealing the slide, it was observed under the microscope [Leica DM4B with Leica Application Suite X (LAS X) software, Wetzlar, Germany]. The number of myelinated nerve fibers per unit area in each group was manually counted.

### Weighing and histological observation of the effector muscle

The gastrocnemius and tibialis anterior muscles of the model and opposite sides were collected from SD rats and weighed. The ratio of the wet weight of the model side muscle to that of the opposite side muscle was calculated. After trimming, the muscle was placed in 4% paraformaldehyde for fixation overnight, and then dehydrated successively with 50, 70, 90, and 100% ethanol. The muscle was embedded in paraffin, and sliced into a 7 μm-thick piece. Then Masson staining was performed. Those for Masson staining were treated with Weigert stain for 8 min and rinsed with distilled water. The sections were then treated with spring red acid complex red for 5 min, immersed in acetic acid for 1 min, treated with phosphomolybdic acid for 2 min, and immersed in acetic acid for 1 min. Next, the sections were treated with aniline blue for 2 min, immersed in acetic acid for 1 min, and rinsed with water. After sealing the slide, it was imaged under a microscope [Leica DM4B with Leica Application Suite X (LAS X) software, Wetzlar, Germany]. The cross-sectional area of gastrocnemius and anterior tibial muscle fibers was calculated using Image J software. Three visual fields of each sample were selected, and the average value was taken as the ultimate muscle fiber cross-sectional area of the sample.

### Retrograde tracing of neurons

Three SD rats were taken from each group for the tracing and labeling experiment, and the method proposed in (12) was adopted. The regenerated sciatic nerve of rats was re-exposed and cut off. The distal ends of the common peroneal nerve and tibial nerve were immersed in 5% Fluoro-Ruby (FR, Life Technologies) and 4% Fluoro-Gold (FG, Life Technologies) for 2 h, respectively. The wound was washed and sutured layer by layer. Then rats were fed for 7 days so that the retrograde tracer could label the spinal cord neurons. After anesthetizing, the heart of rats was isolated and perfused. L4–L6 lumbar spinal cord segments containing sciatic nerves were cut off and fixed in 4% paraformaldehyde for 12 h. The sample was dehydrated in 10, 20, and 30% sucrose for 12 h, and then sliced to a 40 μm-thick piece by a frozen slicer (Leica CM 1950, Germany). Subsequently, the sample was placed on the glass slide and sealed, and the slice was imaged by the 20 × microscope objective. The excitation wavelength was FG330nm and FR561nm. Finally, the acquired fluorescence image was analyzed using Image J software to detect the neurons labeled by FG only, FR only, and both FG and FR.

### Data analysis

All measurement data were expressed by (mean ± standard deviation). One-way ANOVA was employed to analyze data in different groups, followed by Tukey's *post-hoc* multiple comparison test. SPSS 18.0 was used for statistical processing. *P* < 0.05 meant that the difference was statistically significant.

## Results

### Gross observation of nerve repair

**Figure 2:**
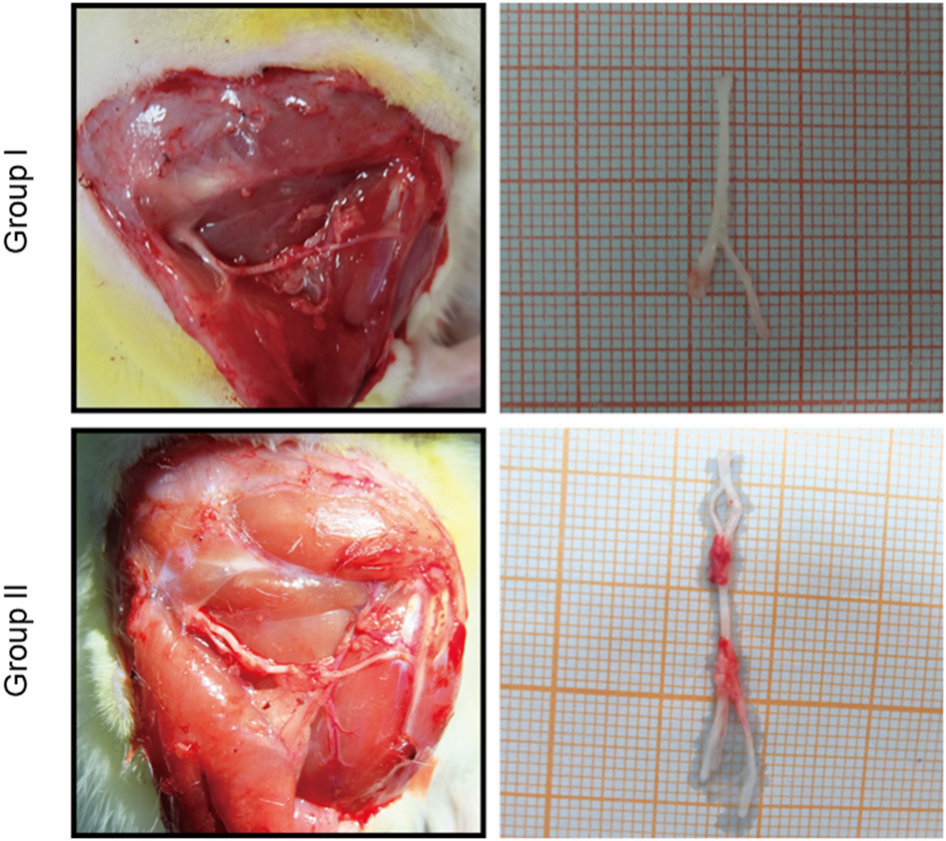
*In vivo* and *in vitro* specimen observation of the repaired nerve. In Group I, only the distal end of the common peroneal nerve was sutured; in Group II, both proximal and distal ends of the common peroneal nerves were sutured.

The proximal and distal common peroneal nerves at the repair site were observed *in vivo* and *in vitro*. In Group I, only the distal common peroneal nerve remained intact, while in Group II, both the proximal and distal ones were complete (Figure 2).

### Sciatic functional index for nerve repair detection

**Figure 3:**
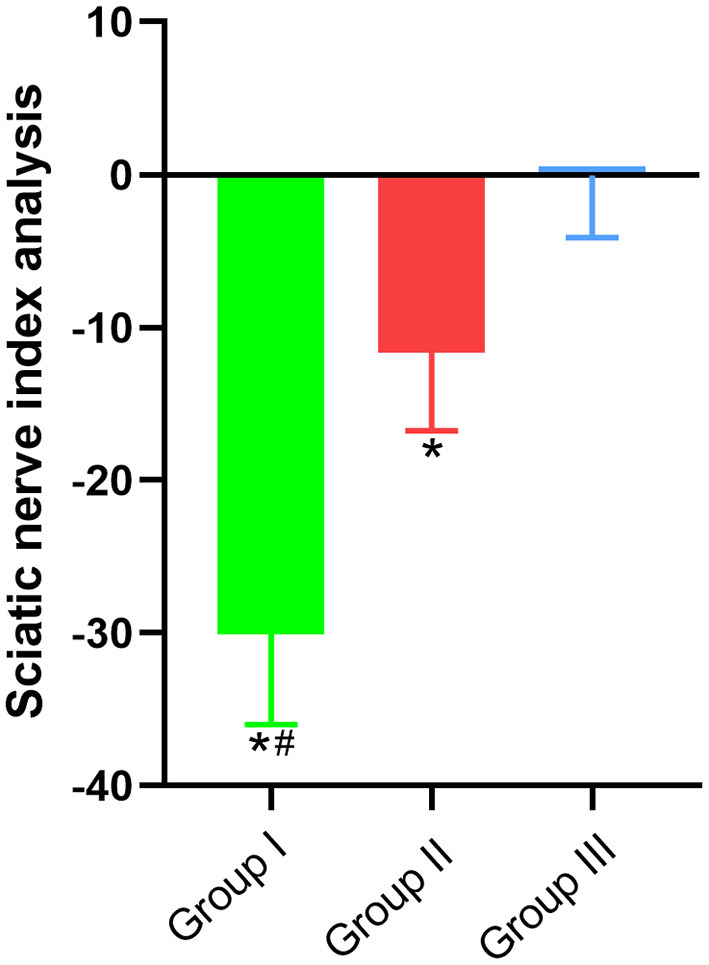
Sciatic functional index. In Group I, only the distal end of the common peroneal nerve was fostered; in Group II, both proximal and distal ends of the common peroneal nerve were fostered; Group III was sham operated normal group. Data are expressed as mean ± SD (*n* = 6 in Group I, 6 in Group II and 6 in Group III). *P < 0.05, vs. Group III; ^#^*P* < 0.05, vs. Group II.

Since the common peroneal nerve and tibial nerve constitute the sciatic nerve, the sciatic functional index can be used to detect the recovery of nerve function. In this paper, the regeneration and repair of the common peroneal nerve were analyzed using the sciatic functional index. The results showed that the sciatic functional indexes of Group I, Group II and Group III were (−30.09 ± 5.9, *n* = 6), (−11.64 ± 5.12, *n* = 6), and (−0.05 ± 4.09, *n* = 6), respectively. Significant statistical difference was found (*p* < 0.05). The sciatic functional index of Group II was better than that of Group I and closer to that of Group III (Figure 3).

### Electrophysiological detection of functional recovery of repaired nerves and their innervating effectors

**Figure 4:**
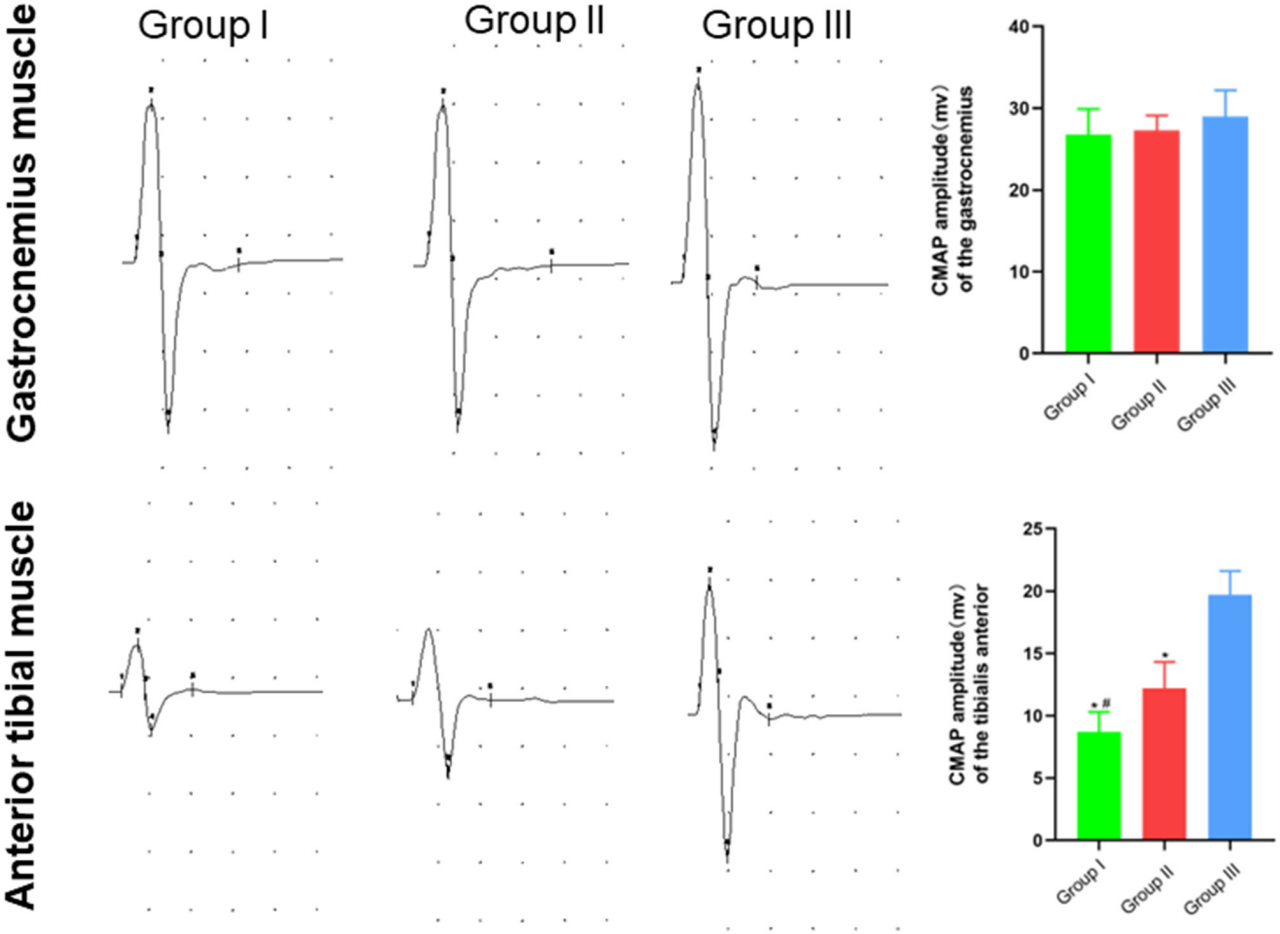
Waveform and amplitude of the compound action potential of gastrocnemius and tibialis anterior muscles detected by electrophysiology. In Group I, only the distal end of the common peroneal nerve was fostered; in Group II, both proximal and distal ends of the common peroneal nerves were fostered; Group III was sham operated normal group. Data are expressed as mean ± SD (*n* = 6 in Group I, 6 in Group II and 6 in Group III). *P < 0.05, vs. Group III; ^#^*P* < 0.05, vs. Group II.

In clinical practice, electromyography is often used to record the electrophysiological characteristics of nerves and muscles. In this study, the functional recovery of the repaired nerves and their innervating effectors were detected by electrophysiology. The CMAP size depends on the number of functional muscle fibers. The detection results suggested that the waveforms of gastrocnemius and tibialis anterior muscles in Group I, Group II, and Group III were not different. The CMAP amplitudes of the gastrocnemius muscle in Group I, Group II and Group III were (26.80 ± 3.10) mV, (27.30 ± 1.80) mV, and (29.00 ± 3.20) mV, respectively, and no significant difference was found (*p* > 0.05). The CMAP amplitude of the tibialis anterior muscle in Group I, Group II and Group III was (8.70 ± 1.60) mV, (12.20 ± 2.10) mV, and (19.70 ± 1.90) mV, respectively, and there was significant statistical difference (*p* < 0.05). The CMAP amplitude of the tibialis anterior muscle in Group II is closer to Group III than Group I is to Group III (Figure 4).

Stimulation electrodes were placed at different positions to check whether the proximal end of the common peroneal nerve was fostered on the tibial nerve, and whether the proximal common peroneal nerve could control the anterior tibial muscle or even the gastrocnemius muscle. According to the results, stimulating point A could cause the contraction of gastrocnemius and anterior tibial muscles in Group I and Group II. When stimulating point B, the gastrocnemius and anterior tibial muscles in Group II tightened. The gastrocnemius and anterior tibial muscles in Group I and Group II constricted as the point C was stimulated. Stimulating point D could lead to the contraction of the gastrocnemius muscle in Group I, Group II and Group III. When the point E was stimulated in Group I, Group II and Group III, the anterior tibial muscle tightened (Table 1).

**Table 1:** Contraction of gastrocnemius/tibialis anterior muscles after stimulation of different positions of nerves.

**Position**	**A**	**B**	**C**	**D**	**E**
Group I	+/+	–/–	+/+	+/–	–/+
Group II	+/+	+/+	+/+	+/–	–/+
Group III	+/–	–/+	+/–	+/–	–/+

“+” means there is muscle contraction activity, and “–“ means no muscle contraction activity; The front of “/” stands for the contraction of the gastrocnemius muscle, and the back of “/” represents the contraction of the tibialis anterior muscle.

### Osmium acid staining for detecting the recovery of peripheral nerves at the repair site

**Figure 5:**
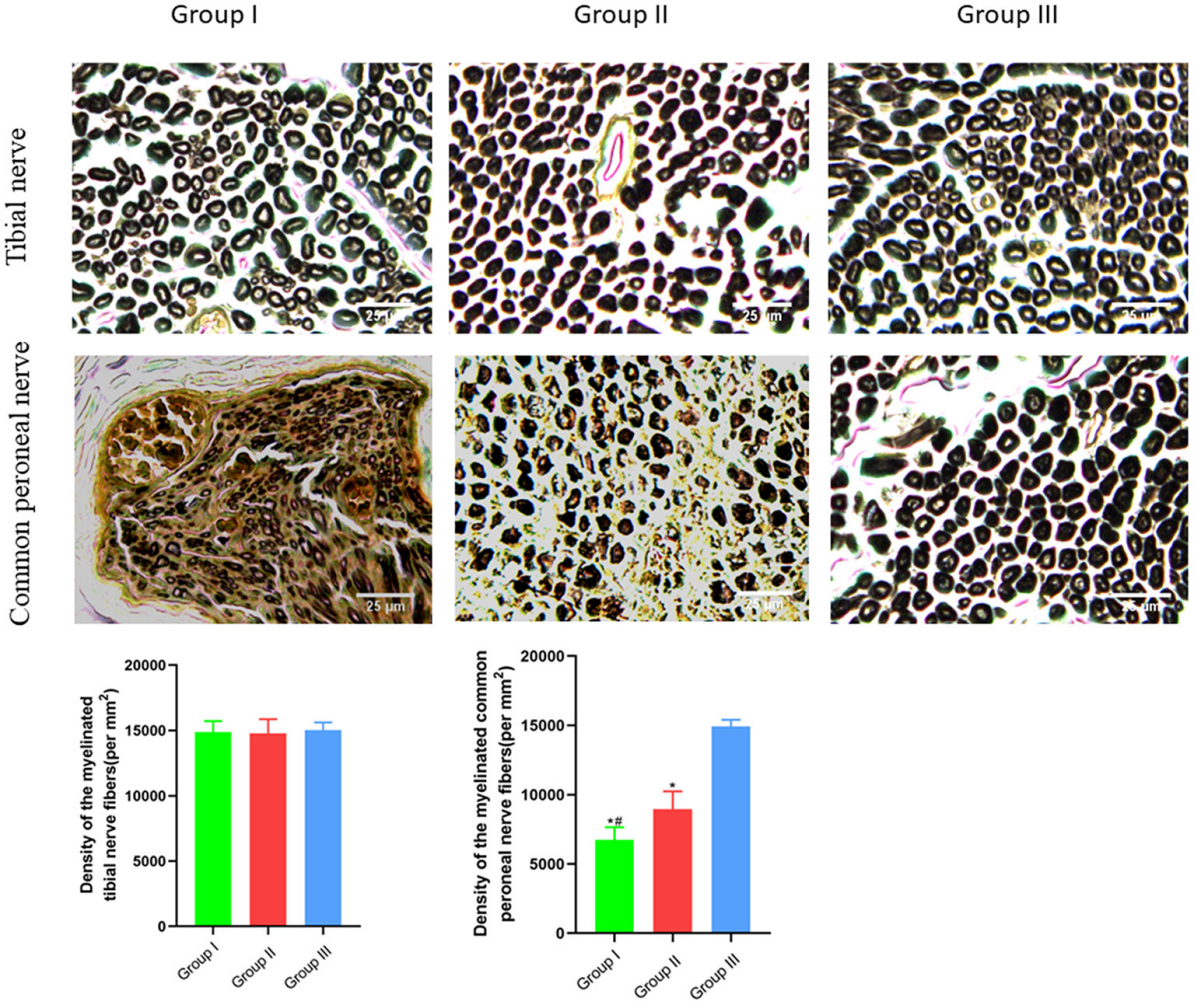
Observation of myelinated tibial and common peroneal nerve fibers per unit area. Osmic acid staining, scale bar = 25 μm. In Group I, only the distal end of the common peroneal nerve was sutured; in Group II, both proximal and distal ends of the common peroneal nerve were repaired; Group III was sham operated normal group. Data are expressed as mean ± SD (*n* = 6 in Group I, 6 in Group II and 6 in Group III). *P < 0.05, vs. Group III; ^#^*P* < 0.05, vs. Group II.

The numbers of myelinated tibial and common peroneal nerve fibers per unit area were calculated using osmium acid staining in each group. The number of myelinated tibial nerve fibers per unit area in Group I, Group II and Group III was (14,883 ± 835) mm^2^/piece, (14,783 ± 1,091) mm^2^/piece and (15,017 ± 608) mm^2^/piece, respectively, and no significant difference is observed (*p* > 0.05). The number of myelinated common peroneal nerve fibers per unit area in Group I, Group II and Group III was (6,750 ± 903) mm^2^/piece, (8,967 ± 1,269) mm^2^/piece, and (14,917 ± 488) mm^2^/piece, respectively, and there was significant statistical difference (*p* < 0.05). The number of myelinated common peroneal nerve fibers per unit area in Group II is closer to Group III than Group I is to Group III (Figure 5).

### Macroscopic and microscopic observation and detection of effector muscle function

**Figure 6:**
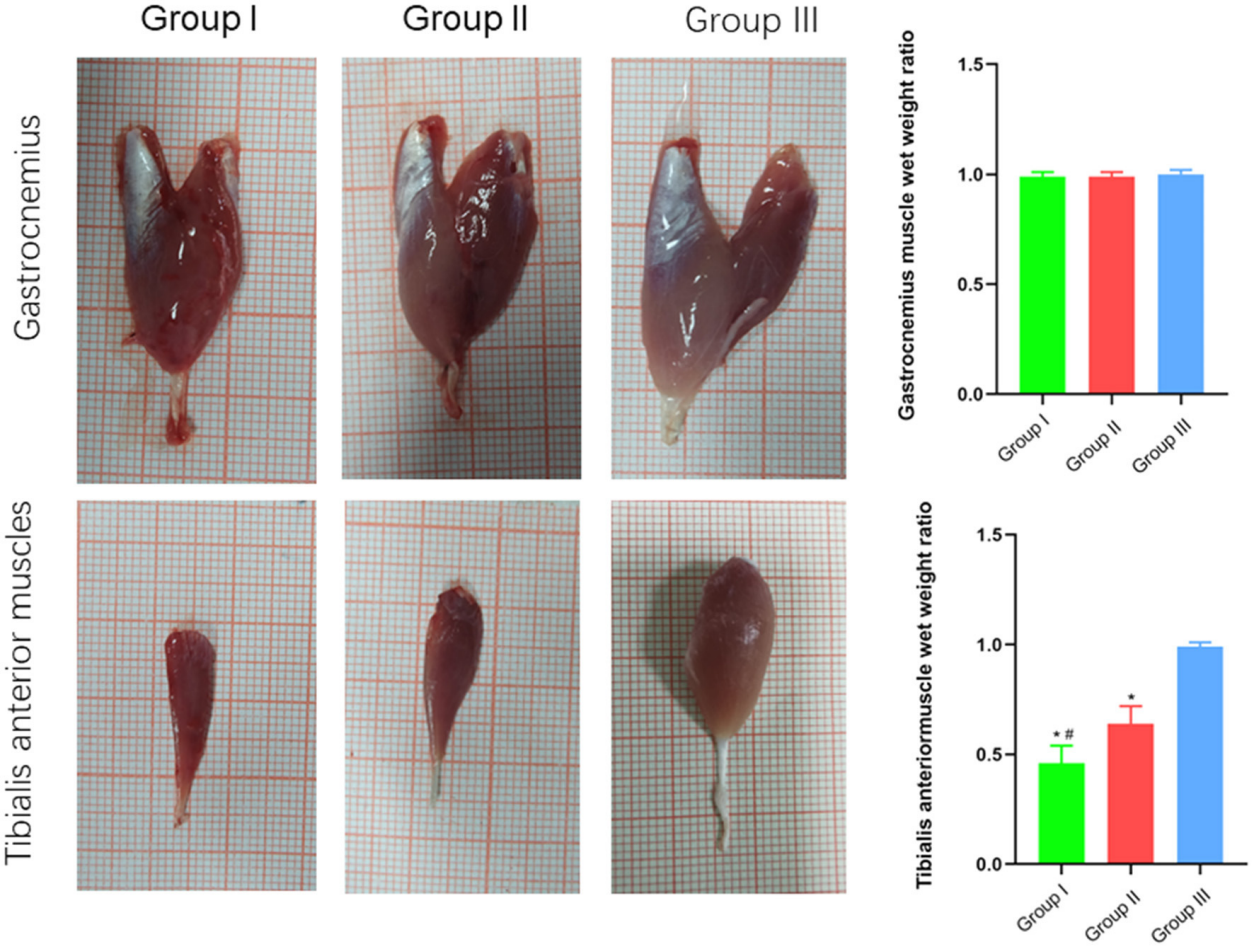
Macroscopic specimen observation of gastrocnemius and tibialis anterior muscles. In Group I, only the distal end of the common peroneal nerve was repaired; in Group II, both proximal and distal ends of the common peroneal nerve was repaired; Group III was sham operated normal group. Data are expressed as mean ± SD (*n* = 6 in Group I, 6 in Group II and 6 in Group III). *P < 0.05, vs. Group III; ^#^*P* < 0.05, vs. Group II.

**Figure 7:**
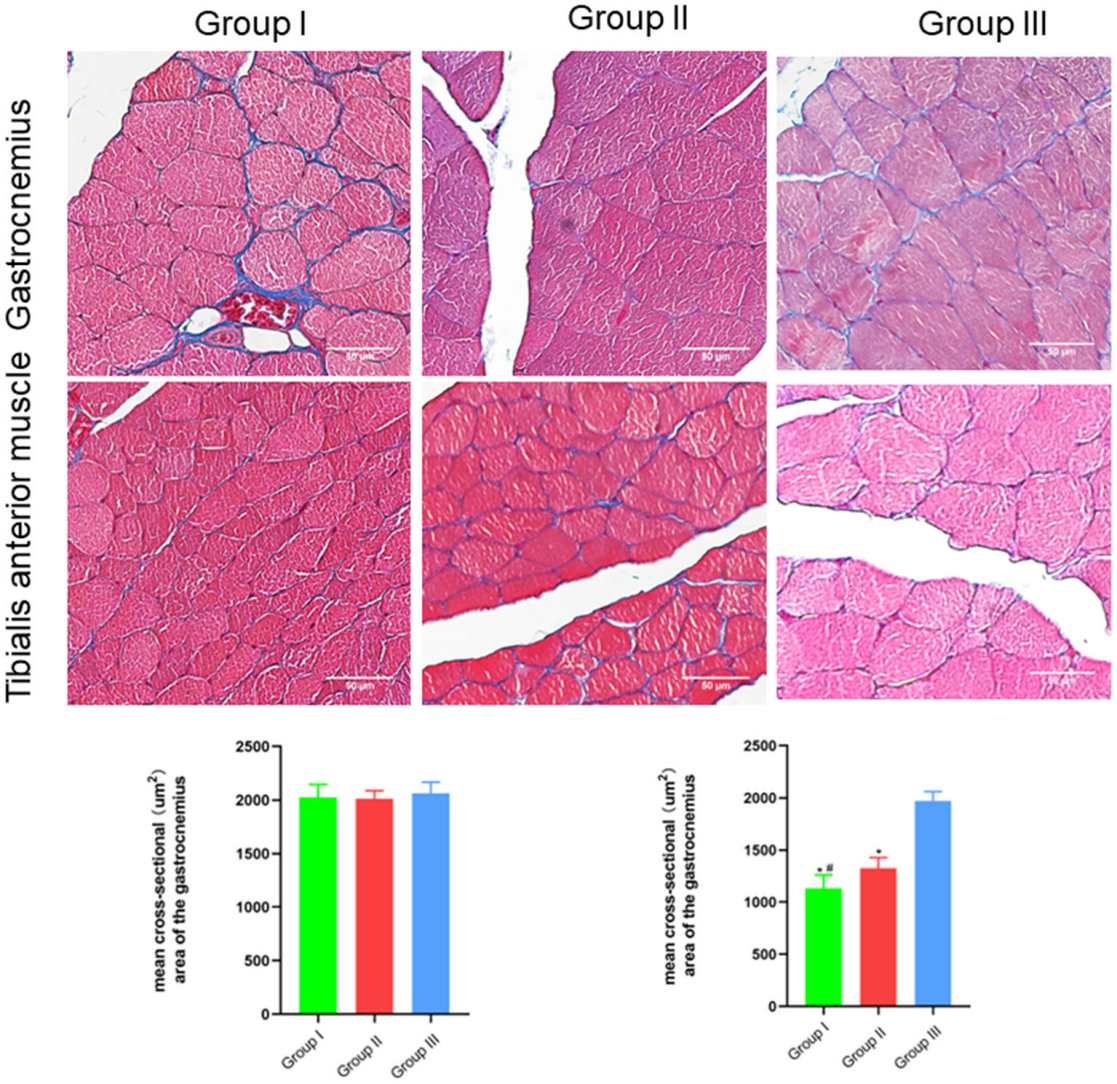
Microscopic specimen observation of gastrocnemius and tibialis anterior muscle. Masson stain, scale bar = 50 μm. In Group I, only the distal end of the common peroneal nerve was repaired; in Group II, both proximal and distal ends of the common peroneal nerve were repaired; Group III was sham operated normal group. Data are expressed as mean ± SD (*n* = 6 in Group I, 6 in Group II and 6 in Group III). *P < 0.05, vs. Group III; ^#^*P* < 0.05, vs. Group II.

The wet weight ratio and the cross-sectional area of gastrocnemius and tibialis anterior muscle fibers were measured using gross specimen and Masson staining methods. The wet weight ratio of gastrocnemius muscle in Group I, Group II and Group III was (0.99 ± 0.02), (0.99 ± 0.02), and (1.00 ± 0.02), respectively. The cross-sectional area of gastrocnemius muscle fibers was (2,025 ± 121) μm^2^, (2,011 ± 76) μm^2^, and (2,061 ± 107) μm^2^, respectively. Both the wet weight ratio and fiber cross-sectional area of gastrocnemius muscle were not significantly different between Group I and Group II (*p* > 0.05). The wet weight ratio of tibialis anterior muscle in Group I, Group II and Group III was (0.46 ± 0.08), (0.64 ± 0.08), and (0.99 ± 0.02), respectively. The cross-sectional area of tibialis anterior muscle fibers was (1,131 ± 130) μm^2^, (1,325 ± 103) μm^2^, and (1,971 ± 90) μm^2^, respectively. Significant statistical difference was found between Group I and Group II in the wet weight ratio and fiber cross-sectional area of gastrocnemius muscle (*p* < 0.05). The wet weight ratio and fiber cross-sectional area of tibialis anterior muscle in Group II is closer to Group III than Group I is to Group III (Figures 6, 7).

### Analysis of the relationship between the repair nerve and motor neurons in the anterior horn of the spinal cord by retrograde tracing of neurons

**Figure 8:**
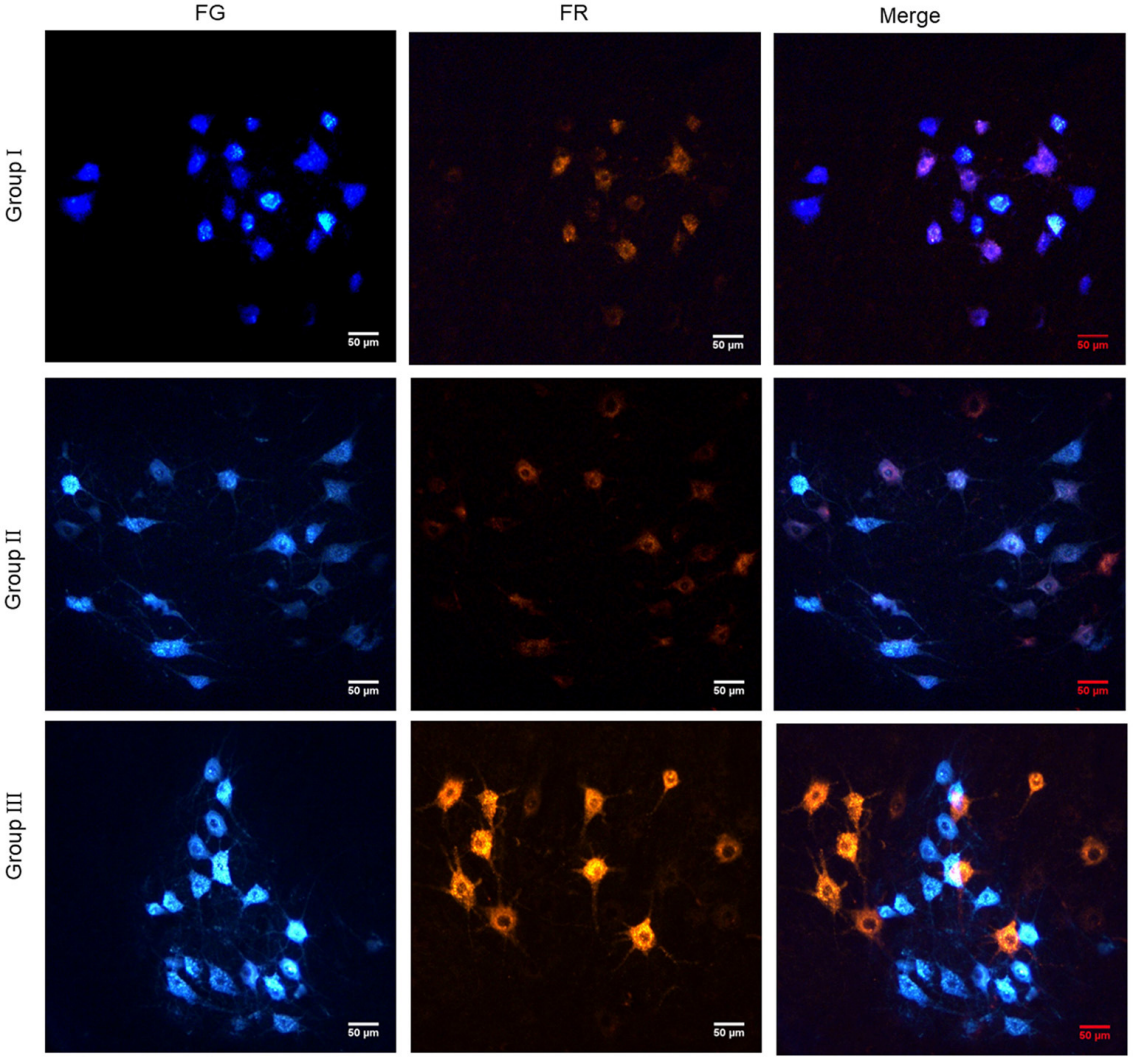
Retrograde tracing of neurons showed the reinnervation connection between neurons and effectors. In Group I, only the distal end of the common peroneal nerve was fostered; in Group II, both proximal and distal ends of the common peroneal nerve were fostered; Group III was sham operated normal group.

Retrograde tracing of neuron was carried out to explore the reinnervation connection between neurons and effectors. There were only FG-labeled, both FG- and FR-labeled fluorescent neurons in the spinal cord of Group I. But there were no only FR-labeled fluorescent neurons. In Group II, only FG-labeled, only FR-labeled and both FG- and FR-labeled fluorescent neurons were observed in the spinal cord. In Group III, only FG-labeled and only FR-labeled fluorescent neurons were found in the spinal cord, but there were no fluorescent neurons labeled by both FG and FR (Figure 8).

## Discussion

Cut, pull and compression induced peripheral nerve injuries can bring about motor, sensory and nutritional dysfunction in the innervated area, and patients may be disabled as a result of delayed treatment (13, 14). The clinical treatment effect for a large segmental peripheral nerve defect is poor, which has become a thorny problem for orthopedic doctors (15, 16). End-to-side anastomosis, first proposed by foreign scholars in 1901, is one method to repair peripheral nerve injuries. This method anastomoses the distal end of the injured nerve with an adjacent normal nerve (17, 18), while the injured proximal end is left open to restore the function of effectors. However, the application of this method is restricted because it wastes the injured proximal peripheral nerve and is prone to cause neuromas and other complications. To overcome the above shortcomings, both the proximal and distal ends of injured nerves should be attached to normal peripheral nerves. In this study, an SD rat model with a large segmental common peroneal nerve defect was established, and end-to-side anastomosis was employed to suture the proximal and distal stumps of the damaged common peroneal nerve to an adjacent tibial nerve. Twelve weeks after repair, the functional recovery of the injured nerve and its effector was assessed, and the connection between the repaired nerve and motor neurons in the anterior horn of the spinal cord was analyzed.

The functions and mechanisms were explored at neuroanatomy and physiology levels. The analysis results showed that when the proximal and distal stumps of the injured common peroneal nerve were simultaneously coapted to the adjacent normal tibial nerve, both ends remained intact without neuromas detected. This finding suggests that to repair the proximal common peroneal nerve is better than to keep the proximal common peroneal nerve open. The tibialis anterior muscle and gastrocnemius muscle are innervated by the common peroneal nerve and tibial nerve, respectively. According to the experimental results of this study, no matter whether the proximal end of the common peroneal nerve was fostered or not, the ipsilateral tibialis anterior muscle and gastrocnemius muscle constricted when the tibial nerve near the distal foster point of the common peroneal nerve was stimulated. When the proximal end of the common peroneal nerve was sutured, stimulating the proximal end of the common peroneal nerve could also make the ipsilateral tibialis anterior and gastrocnemius muscles contract. However, when the distal end of the common peroneal nerve was open, two muscles did not tighten. As for the CMAP, it was found that the compound action potential of the gastrocnemius muscle when stimulating the distal foster point of the common peroneal nerve or the open proximal end was not significantly different from that when stimulating the normal nerve at the same level. This result remains true no matter whether the proximal end of the common peroneal nerve was repaired or not. The compound action potential of the tibialis anterior muscle in the foster group was higher than that in the open group and closer to that in the normal group. The wet weight ratio and cross-sectional area of the gastrocnemius muscle in the three groups had no significant difference. The wet weight ratio and cross-sectional area of the tibialis anterior muscle in the foster group were greater than those in the open group and closer to those in the normal group. Similar results were obtained in detecting the recovery of the tibial nerve and the common peroneal nerve 2 cm below the distal foster nerve of the same height in the three groups. There was no significant difference in the number of myelinated tibial nerve fibers per unit area among the three groups. The number of myelinated common peroneal nerve fibers per unit area in the foster group was more than that in the open group and closer to that in the normal group. Previous studies have confirmed (19, 20) the importance of repairing proximal nerves in the treatment of peripheral nerve injuries. The reason may be that more nerves are available for attracting peripheral repair materials, and donor nerve epineuria act as a bridge for axon regeneration. In this process, proximal nerve endings can grow directly to the distal end, and serve as a catheter to allow the peripheral repair materials to reach the distal injury site through the donor nerve adventitia (11). A similar effect was observed in the present research. When the proximal and distal common peroneal nerves were injured and sutured simultaneously to the peripheral normal tibial nerve, the conditions of injured nerves and corresponding effectors after repair were better than those when only the injured distal common peroneal nerve was sutured. This may be due to the secretion of such endogenous substances as nerve growth factors (21), basic fibroblast growth factors (22), and glial cell derived neurotrophic factors (23) around the injured nerve through relevant signaling pathways (e.g., PI3K/AKT, Notch, TAK1-MAPK/NF-κB etc.) (24–26). These factors affect the function of Schwann cells (27) or macrophages (28) and promote the repair of injured peripheral nerves. During the proximal foster caring process, abundant endogenous factors can further regulate relevant signaling pathways and act on cells around the injured proximal and distal nerves through the bridge between the outer membrane of the donor and the distal end. A benign cycle is thus formed, which helps accelerate the repair process and promote the protection and recovery of effector functions. Bontioti et al. (29) believed that fostering both the proximal and distal stumps on peripheral normal nerves was not beneficial to the recovery of the injury effector function, but this does not affect the interpretation of the results of this research. In the study of Bontioti, the rat model of brachial plexus injury was used. Since the rat's forepaw has more complex functions than its hind paw, damage repair is more difficult. However, the specific mechanism deserves further investigation.

The axon has the ability of collateral regeneration (30–32). In this paper, the axon was also proven capable of reinnervating the recipient nerve and effector through collateral sprouting during end-to-side anastomosis. Especially when both the proximal and distal ends were anastomosed, the proximal common peroneal nerve could reinnervate the distal common peroneal nerve, tibial nerve and effector. The improved end-to-side anastomosis method proposed in this paper provides more donor nerves, so the nerve function recovers better. This research has some limitations. Firstly, previous studies have confirmed that with the extension of repair time, only one axon sprouting from the lateral branch can be retained (12, 33). In this study, when both the injured proximal and distal nerves are hosted on the peripheral nerves, the axons sprouting from the lateral branch and especially, the neurons with multiple axons, can maintain their functions. Nevertheless, this result requires further validation. Secondly, in this paper, the molecular mechanism for injured distal nerve and effector repair by coapting the injured proximal and distal nerves simultaneously to the peripheral nerves is analyzed based on only previous study findings. The specific mechanism needs to be verified through molecular biology experiments.

## Conclusion

An effective method to repair injured peripheral nerves with a large segmental defect is to anastomose both the injured proximal and distal ends to the normal peripheral nerve. To repair the injured proximal peripheral nerve is also an important factor to recover the function of injured nerve. The injured nerves can be innervated by establishing a connection with the distal effector through the fostered nerve.

## Data availability statement

The original contributions presented in the study are included in the article/supplementary material, further inquiries can be directed to the corresponding authors.

## Ethics statement

The animal study was reviewed and approved by Medical Ethics Committee of Peking University People's Hospital.

## Author contributions

DL, QY, FY, and JP designed the experiments. DL, QY, FY, XL, JJ, GL, KB, and SJ carried out the experiments. FY and JP supervised the whole experimental process and revised the manuscript. XL, JJ, GL, KB, and SJ analyzed the data. DL and QY wrote the manuscript. All authors contributed to the article and approved the submitted version.
